# Coping and emotions of global higher education students to the Ukraine war worldwide

**DOI:** 10.1038/s41598-024-59009-3

**Published:** 2024-04-12

**Authors:** Daniela Raccanello, Roberto Burro, Aleksander Aristovnik, Dejan Ravšelj, Lan Umek, Giada Vicentini, Rob Hall, Chiara Buizza, Muhammad Ayub Buzdar, Surobhi Chatterjee, Nicola Cucari, Beata Dobrowolska, Ana Teresa Ferreira-Oliveira, Thais França, Alberto Ghilardi, Fany Inasius, Sujita Kumar Kar, Konstantinos Karampelas, Andrii Kuzyshyn, Florin Lazăr, Juan D. Machin-Mastromatteo, Maria Malliarou, Bertil P. Marques, Silvia Mariela Méndez-Prado, Cristina Mollica, Alka Obadić, Olawale Festus Olaniyan, Ana Sofia Rodrigues, Giulio Sbravati, Aleksandra Vasić, Ana-Maria Zamfir, Nina Tomaževič

**Affiliations:** 1 Department of Human Sciences, University of Verona, Verona, Italy; 2 Faculty of Public Administration, University of Ljubljana, Ljubljana, Slovenia; 3 Department of Psychology, Macquarie University, Sydney, Australia; 4 Environmetrics, Killara, Australia; 5 Department of Clinical and Experimental Sciences, University of Brescia, Brescia, Italy; 6 Department of Secondary Teacher Education, Allama Iqbal Open University Islamabad, Islamabad, Pakistan; 7 Department of Psychiatry, King George’s Medical University, Lucknow, India; 8 Department of Management, Sapienza University of Rome, Rome, Italy; 9 Faculty of Health Sciences, Medical University of Lublin, Lublin, Poland; 10 CISAS, Polytechnic Institute of Viana Do Castelo, Viana do Castelo, Portugal; 11 Centre for Research and Studies in Sociology, Cies-Iscte, Lisbon, Portugal; 12 School of Accounting, Bina Nusantara University, Jakarta, Indonesia; 13 Pedagogic Department of Elementary Education, University of the Aegean, Mytilene, Greece; 14 Ternopil Volodymyr Hnatiuk National Pedagogical University, Ternopil, Ukraine; 15 Faculty of Sociology and Social Work, University of Bucharest, Bucharest, Romania; 16 Faculty of Philosophy and Letters, Autonomous University of Chihuahua, Chihuahua, Mexico; 17 Department of Nursing, University of Thessaly, Vólos, Greece; 18 Institute of Engineering of Porto, P.PORTO-Polytechnic of Porto, Porto, Portugal; 19 Faculty of Social Sciences and Humanities, ESPOL Polytechnic University, Guayaquil, Ecuador; 20 Department of Statistical Sciences, Sapienza University of Rome, Rome, Italy; 21 Faculty of Economics and Business, University of Zagreb, Zagreb, Croatia; 22 School of Agriculture and Environmental Sciences, University of The Gambia, Serrekunda, Gambia; 23 Faculty of Law, University of Niš, Niš, Serbia; 24 National Scientific Research Institute for Labour and Social Protection, Bucharest, Romania

**Keywords:** Psychology, Human behaviour

## Abstract

Trauma scientists have raised the alarm about the devastating consequences of the Ukraine war on mental health. We examined how higher education students—as indirect victims—coped with this conflict and how they emotionally reacted during 2022. We involved 2314 students from 16 countries through an online survey. A structural equation model indicated significant relations between war-related worry about military and macroeconomics domains and two coping strategies (opposition, support giving), in turn significantly linked with six emotions. The model was strongly invariant across gender, study field, and geographic area. The most frequent emotions were anger and anxiety, followed by two future-centred emotions (hopelessness and hope). Emotions were more frequent for females and students of the countries geographically close to the war region. Our findings call for evidence-based policy recommendations to be implemented by institutions to combat the negative short and long-term psychological sequelae of being witnesses of armed conflicts.

## Introduction

When Russia invaded Ukraine on 24 February 2022, it gave rise to a war whose economic, social, and psychological traumatic impact still reverberates worldwide^1^. Within the first 3 months of the war, the Office of the United Nations High Commissioner for Human Rights reported more than 4000 deaths and almost 5000 injured people among civilians in Ukraine while acknowledging that the actual figures were probably significantly higher^2^. In the same period, the Russian invasion caused 6.3 million people, mainly children, women, and the elderly, to flee from Ukraine^3,4^. In an attempt to end the aggression, more than 30 countries imposed sanctions on Russia. However, even in those first months, the sanctions’ effects were uncertain and debated at individual, economic, and diplomatic levels^1,5^. In parallel, there were extraordinary solidarity reactions in Europe and beyond regarding material and psychological support towards refugees^1,6^.

Knowing more about how people, directly or indirectly affected by war, cope with the traumatic effects on physical and psychological health is important for developing successful ways to support those who suffer.

Increased psychopathology amongst adults involved in wars and armed conflicts has been well documented, with increased rates of anxiety, depression, and post-traumatic stress disorder (PTSD)^7–9^. War-related trauma might even be transmitted through biological and psychological mechanisms, resulting in long-term effects for more than one generation^10,11^. Trauma specialists have raised concerns about the potentially devastating consequences of the current war on the mental health of people involved in Ukraine, Russia, and more generally across the globe^10,12^. Involving direct and vicarious war victims, Chudzicka-Czupała et al.^13^ conducted an online survey with 12 to 49-year-old participants during March and April 2022, indicating higher levels of depression, anxiety, and stress for Ukrainians people compared to Poles, for whom they were, in turn, higher than for Taiwanese participants. Some studies conducted in Ukraine during 2022 focussed on Higher Education (HE) students, detecting also for this population increases in PTSD, depression, exhaustion, loneliness, nervousness, and anger, with higher levels among females than males^12,14^. These outcomes, together with the growth of public concern about posttraumatic symptoms amongst Ukrainians^15^ and the number of hospital admissions from war-related traumas, occurred when mental health services in Ukraine were diminishing^16^.

It is not only people directly exposed to war who can suffer psychologically—experts refer to secondary or vicarious traumatisation or victimisation^17,18^. Even though the literature addressing the indirect impact of war on HE students is small, students’ reactions to violent acts such as terror attacks have been partially investigated. For example, some studies revealed increases in anxiety, PTSD, distress, and negative affective states due to indirect media exposure to terror attacks for Israeli, American, and Italian university students^17,19–22^. However, most students’ symptoms appear to diminish over time^20,23^.

Two of the many ways people can cope with stressful and traumatic events are opposing others or supporting others. The psychological literature has frequently documented the maladaptive impact of oppositional responses on mental health and well-being, contrasting them with the adaptive function of social support^24,25^. However, not much is known about how these processes play out in the context of war and amongst indirect victims—i.e., those who are not directly involved in the conflict but are, for example, in contact with direct victims or by being exposed to reports in the mass media^26^. Deepening our understanding of active coping strategies—such as opposition or support giving—and their emotional correlates is a prerequisite to developing programs fostering the most adaptive reactions to traumatic events for both direct and indirect victims. This can be particularly relevant for HE students, who easily access the news, especially through the Internet and social media^27^.

Therefore, we examined the relationship between how HE students coped with the Ukraine war and their emotional reactions—as indirect victims—during the Northern Hemisphere spring of 2022. The participants in our survey were HE students from 16 countries that had not been directly involved in the conflict.

Coping refers to an individual’s cognitive, emotional, and behavioural responses when facing stressful events^28,29^. Such efforts constantly vary as a function of changes in the person-environment relationship^30^. These changes can partially result from coping processes aimed at modifying the situation (i.e., problem-focused coping) or distress (i.e., emotion-focused coping), from changes pertaining to the person, or from variations in the context independent of the individual. The latter can include a range of domains, such as, in the case of a war, the educational, economic, or military domains. Given the potential strength of contextual conditions to influence people’s reactions, coping and the related emotions are highly variable among and within people^30^. Therefore, it is important to study them in specific contexts.

The literature on coping has long debated the reciprocal influences between coping and emotions, and experts agree on their dynamic mutual reciprocal relations^30^. Coping can be conceptualised as a mediator between emotional states. The flow begins with the individual appraising something as a threat. This, in turn, triggers a coping mechanism, which may cause a change in the person-environment relationship leading to an emotional response^30^. So, when HE students are indirectly exposed to war, they may experience increased worry about various aspects of their life. This can affect how they cope, resulting in negative or positive emotions.

What do we already know about how HE students not directly involved in wars and armed conflicts cope? What do we know about the links between their coping and their emotions? Some hints could be drawn from the terrorism literature about how HE students coped. In those contexts, it is generally acknowledged that problem-focused strategies positively affect mental health and emotion-focused strategies negatively impact mental health; however, findings are sometimes inconsistent^17,19,20,23,31,32^. Similar inconsistencies emerged from a study comparing Ukrainian, Polish, and Taiwanese respondents’ coping during the first months of the Ukraine war^13^. Problem-focused coping strategies were helpful in dealing with depression, anxiety, and stress for Polish participants; moreover, they seemed to reduce hopelessness for Ukrainians, were positively related for Poles, and unrelated for Taiwanese. To help disambiguate these effects, we studied a group with indirect war experience (i.e., HE students not directly involved in the conflict). We also examined the impact of geographical distance from the active fighting and demographic variables such as gender and field of study. We focused on two specific types of problem-focused active coping strategies that people indirectly exposed to wars might adopt: opposition and giving social support. In doing so, we sought to help resolve previous inconsistent findings, possibly due to variation within and among sample populations and their use of coping strategies^32^.

The role of social support has already been explored in studies on the impact of the Ukraine war. Prati^5^ examined some psychological factors about the European Union’s (EU) measures in response to Russia’s invasion by analysing the data of more than 26,000 EU citizens (Flash Eurobarometer 506^33^). He found that the tendency to provide humanitarian support to Ukrainians fleeing the war was predicted by feelings of sympathy towards them, in line with the empathy-altruism hypothesis, according to which empathic concern stimulates altruistic motivation^34^. Moreover, their support for the sanctions was related to how EU citizens perceived Ukrainians as part of their community, as assumed by the theory of psychological sense of community^35^. Politi et al.^36^ conducted research amongst French-speaking adults in Belgium. They identified two mechanisms underlying intentions to help Ukrainians: One was predicted by dispositional prosociality (through the mediation of empathy towards Ukrainians) and the second by European identification (through the mediation of identity fusion with Ukrainians). However, while single individuals could, in part, implement support as operationalised in these studies, sanctions were not under their control. Therefore, these findings do not help to shed light on the correlates of individual coping in terms of opposing or supporting strategies.

The current research is part of a large-scale online survey entitled “Students’ perception on the Russia-Ukraine War 2022”^37^, promoted by the University of Ljubljana, Slovenia. It aimed to examine HE students’ beliefs and emotions about the impact of the war. For this study, we examined the data of HE students from 16 countries (not including Russia and Ukraine) during the second and third month of the Ukraine war in 2022. We investigated whether and how their war-related worry was associated with war-related coping and, in turn, linked with their emotions. We also explored whether emotions differed according to gender, study field, and geographic area.

We conceptualised different life domains as antecedents of worry by relying on Bronfenbrenner’s bio-ecological theory^38^. It is worth anticipating that worry can be considered as a construct independent from anxiety^39,40^: The first is focused on cognitive aspects while the latter on affective ones. Bronfenbrenner’s model distinguishes contexts in terms of, among the others, three systems—(a) a microsystem, i.e., circumstances in which individuals are directly involved (e.g., pertaining to students’ education); (b) a macrosystem, i.e., elements characterising a culture such as values, traditions, and sociocultural characteristics (e.g., regarding global macroeconomics issues); and (c) a chronosystem, i.e., the set of historical and social events having an impact on the other systems (e.g., such as current historical events like the Ukraine war). While the escalation of a war can have visible effects in all these three systems for people directly exposed to it^41^, in the case of vicarious exposure the more evident changes regard the macrosystem and the chronosystem. However, also indirect victims could experience some negative consequences in the contexts in which they are directly involved (i.e., microsystems). For example, students could be afraid of possible difficulties in their academic future related to general economic threats or limitations in international experiences. Therefore, distinguishing these three systems permits to explore further how vicarious victims react to wars.

Acknowledging that examining the correlates of coping only in terms of psychopathology is reductive, especially when interested in the psychological functioning of people indirectly exposed to wars, and that psychopathological reactions are different from the intense responses that are normative following extraordinary events^31,42^, we focused on HE students’ affective states considering six emotions. These were of negative (anger, shame, anxiety, and hopelessness) and positive (hope and pride) valence. Three of them were present-centred (anger, shame, and pride); and three were prospective and future-centred (anxiety, hopelessness, and hope)^43^, enabling us to examine students’ affective reactions in a moment of great uncertainty concerning the future.

Emotions can also be described by examining how specific events are related to objectives relevant to the individuals^44^. Anger arises when people appraise that an event threatening one’s objectives is unfair; however, there is still space for doing something. This could result from the perception of an aggression as cruel, divisive, and destabilising, as can happen for a war^1,13^. Shame and pride are two moral emotions that might occur from acknowledging one’s faults or merits in relation to the achievement of specific objectives and are connected with the interests or welfare of individuals or society^18,45^; they depend on the extent to which people feel disengaged or engaged in actions valued negatively (e.g., opposing) or positively (e.g., giving support), in this case, with reference to war. Anxiety is the prospective emotion which parallels fear. This emotion typically engages the brain in the ‘fight-or-flight mode’ and becomes particularly intense in case of prolonged events which threaten people’s survival and well-being^46^. Hopelessness belongs to the family of sadness and is related to the awareness of the ineluctability of a loss due to obstacles threatening one’s objectives: Concerning the war, it mirrors the widely diffused concern and despair of billions of people and is linked to a feeling of helplessness^6,10^. Conversely, hope is the positive emotion associated with the possibility that one’s objectives can be achieved in the future.

Notwithstanding the importance of this topic, researchers have rarely addressed these issues using cross-national samples of HE students as indirect victims of war. From a theoretical perspective, this work fills at least four theoretical gaps in the literature by examining: (a) the interactions between active problem-focused strategies (in our case, opposition and support giving) and their emotional correlates, for which previous studies reported contradictory results; (b) the role of antecedents of coping conceptualized using Bronfenbrenner’s perspective as the theoretical framework; (c) how the link between strategies and emotions are manifested in a cross-national sample indirectly exposed to the Ukraine war; and (d) the specific population of HE students. From an applied perspective, knowledge of the correlates of emotions during a war can suggest ways to improve HE students’ reactions and foster adopting more adaptive coping strategies.

We had three main aims. The first aim was to test a model in which worry due to different war-related domains (education, macroeconomics, and military) was linked to war-related coping strategies (opposition and support giving), and in turn linked to six emotions of negative (anger, shame, anxiety, and hopelessness) and positive (hope and pride) valence.

### Hypothesis 1a

We hypothesised that the intensity of war-related worry due to different domains was associated with war-related coping strategies regarding opposition and support giving. We expected that for our participants who were not directly involved in the conflict, worry for dimensions about the microsystem (i.e., education domain) was weakly related to the two coping strategies. Moreover, we expected significant links between worry about the macrosystem (i.e., macroeconomics domain) and the chronosystem (i.e., military domain) on the one hand and coping on the other hand.

### Hypothesis 1b

We hypothesised that students with higher opposition felt negative emotions (anger, shame, anxiety, and hopelessness) more frequently and positive emotions (hope and pride) less frequently, and vice versa for students with higher support giving.

The second aim was to examine the invariance of the model on the relations between war-related worry, war-related coping, and emotions to explore the generality of such relations.

### Hypothesis 2

We expected that the tested model would be invariant across gender (male, female), study field (social sciences, applied sciences, natural and life sciences, and arts and humanities), and geographic area (countries geographically belonging to Europe versus other countries), i.e., that the relations between the variables did not change.

The third aim was to explore differences in the mean scores of emotions according to gender, study field, and geographic area. This aim was exploratory, and we did not formulate any specific hypothesis.

## Results

### Structural equation model (Aim 1)

**Figure 1:**
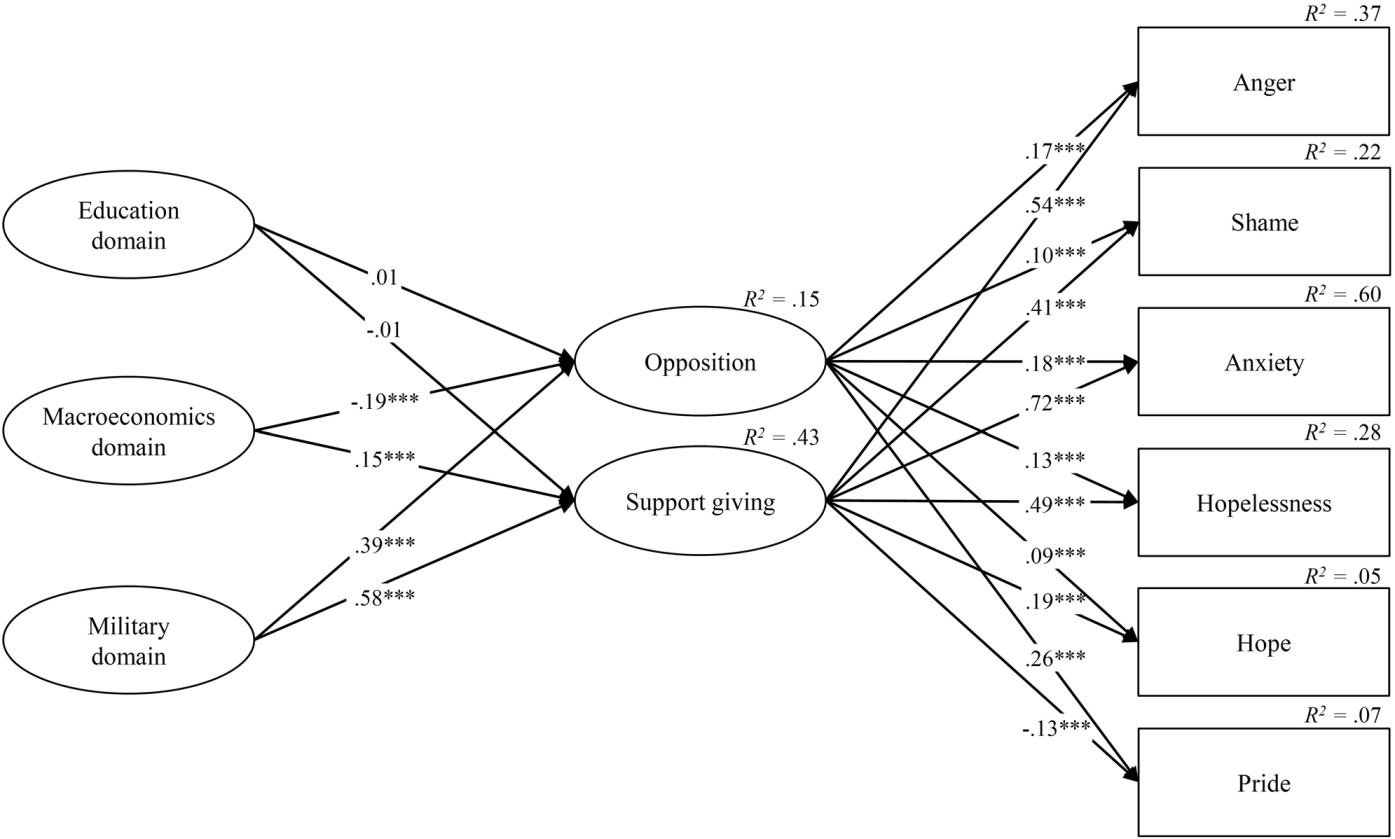
Structural equation model for the relations between war-related worry, coping, and emotions. ***p < 0.001. For parsimony, we represented only the paths related to the structural model (the indexes about the links of both the measurement and the structural model are reported in Table A of the Supplementary Materials).

We ran a structural equation model (SEM) testing the relations between war-related worry (about education, macroeconomics, and military domains) and coping (opposition and support giving), and in turn, between coping and six emotions (anger, shame, anxiety, hopelessness, hope, and pride). We reported the SEM diagram in Fig. 1; intercorrelations and descriptive statistics in Table 1; and factor loadings in Table A of the Supplementary Materials. The loadings were statistically significant at *p* < 0.001, and larger than the 0.45 cut-off value. Reliability was adequate, with omega (ω) ranging from 0.834 (opposition) to 0.912 (education domain). The SEM had a good fit, with χ^2^(269, *N* = 2314) = 3843.197, *p* < 0.001, Tucker Lewis index (TLI) = 0.983, goodness-of-fit index (GFI) = 0.988, root mean square error of approximation (RMSEA) = 0.076, square root mean residual (SRMR) = 0.062, comparative fit index (CFI) = 0.986, and adjusted goodness-of-fit index (AGFI) = 0.981. Most of the structural paths were significant at *p* < 0.001 (except for two non-significant paths between the education domain and coping). The model explained 37% of variance for anger, 22% for shame, 60% for anxiety, 28% for hopelessness, 5% for hope, and 7% for pride.

**Table 1:** Intercorrelations, means (*M*), standard deviations (*SD*), 95% confidence intervals (CI), and McDonald’s omega (ω) for the variables of the SEM.

Variable	1	2	3	4	5	6	7	8	9	10	11
1. Education domain	–						-				
2. Macroeconomics domain	0.519***	–						–			
3. Military domain	0.425***	0.498***	–						–		
4. Opposition	0.057**	0.035	0.240***	–						–	
5. Support giving	0.096***	0.189***	0.320***	0.294***	–						
6. Anger	0.260***	0.256***	0.332***	0.252***	0.316***	–					
7. Shame	0.186***	0.205***	0.206***	0.157***	0.182***	0.444***	–				
8. Anxiety	0.186***	0.316***	0.461***	0.272***	0.302***	0.528***	0.347***	–			
9. Hopelessness	0.174***	0.209***	0.318***	0.191***	0.234***	0.456***	0.438***	0.452***	–		
10. Hope	0.092***	0.089***	0.092***	0.111***	0.159***	0.149*	0.113***	0.197***	0.058**	–	
11. Pride	0.030	− 0.050*	− 0.033	0.179***	− 0.025	0.051*	0.048*	0.091***	0.023	0.354***	–
*M*	2.75	3.59	3.41	2.23	3.68	3.15	2.47	3.08	2.72	2.72	1.78
*SD*	1.20	0.98	1.12	0.93	1.09	1.20	1.29	1.17	1.24	1.14	1.07
95% CI	[2.70, 2.79]	[3.55, 3.63]	[3.37, 3.46]	[2.19, 2.27]	[3.63, 3.72]	[3.11, 3.20]	[2.42, 2.53]	[3.03, 3.13]	[2.67, 2.77]	[2.68, 2.77]	[1.74, 1.83]
ω	0.912	0.879	0.897	0.834	–	–	–	–	–	–	–

*p < 0.05, **p < 0.01, ***p < 0.001.

Concerning the relation between worry-related domains and coping, we found that the education domain was not significantly linked nor with opposition (β = 0.01, *p* = 0.449) neither with support giving (β = 0.01, *p* = 0.819). The macroeconomics domain was significantly related to coping, negatively with opposition (β = -0.19, *p* < 0.001) and positively with support giving (β = 0.15, *p* < 0.001). The military domain was positively connected with both opposition (β = 0.39, *p* < 0.001) and support giving (β = 0.58, *p* < 0.001). These findings partially supported Hypothesis 1a.

In turn, opposition was positively and significantly related to all the emotions (anger: β = 0.17, *p* < 0.001; shame: β = 0.10, *p* < 0.001; anxiety: β = 0.18, *p* < 0.001; hopelessness: β = 0.13, *p* < 0.001; hope: β = 0.09, *p* < 0.001; pride: β = 0.26, *p* < 0.001). Also support giving was significantly linked with all the emotions, negatively with pride (β = -0.13, *p* < 0.001) and positively with all the others (anger: β = 0.54, *p* < 0.001; shame: β = 0.41, *p* < 0.001; anxiety: β = 0.72, *p* < 0.001; hopelessness: β = 0.49, *p* < 0.001; hope: β = 0.19, *p* < 0.001). Our findings corroborated only partially Hypothesis 1b. However, it is worth noting that the significant positive links between coping and emotions were always less intense for opposition than support giving.

### Invariance analysis (Aim 2)

Considering the differences in CFI, we found that the model was characterised by configural, scalar, and metric (strong) invariance for gender (ΔCFI_metric-config_ < 0.001; ΔCFI_scalar- metric_ = 0.001), study field (ΔCFI_metric-configural_ < 0.001; ΔCFI_scalar-metric_ < 0.001), and geographic area (ΔCFI_metric-configural_ = 0.001; ΔCFI_scalar-metric_ = 0.002). Also the exam of the differences concerning the other fit indexes (TLI, RMSEA, and SRMR) confirmed that the model was strongly invariant across each factor (Table 2). Overall, these findings supported Hypothesis 2 and resulted a prerequisite for comparing group means^47^.

**Table 2:** Results of invariance analyses across gender (male, female), study field (social sciences, applied sciences, natural and life sciences, arts and humanities), and geographic area (countries geographically belonging to Europe, other countries).

Groups	Model	χ^2^	df	*p*	TLI	RMSEA	SRMR	CFI	ΔTLI	ΔRMSEA	ΔSRMR	ΔCFI
Gender	Configural invariance	4131.56	538	< 0.001	0.982	0.076	0.066	0.985	–	–	–	–
	Metric invariance	4178.27	553	< 0.001	0.982	0.075	0.066	0.985	< 0.001	0.001	< 0.001	< 0.001
	Scalar invariance	4405.90	626	< 0.001	0.984	0.072	0.066	0.984	0.001	0.003	< 0.001	0.001
Study field	Configural invariance	4444.75	1076	< 0.001	0.985	0.074	0.067	0.987	–	–	–	–
	Metric invariance	4534.77	1121	< 0.001	0.985	0.073	0.067	0.987	< 0.001	0.001	0.0	0.0
	Scalar invariance	4679.56	1340	< 0.001	0.988	0.066	0.067	0.988	0.003	0.007	< 0.001	< 0.001
Country	Configural invariance	3556.52	538	< 0.001	0.986	0.070	0.059	0.989	–	–	–	–
	Metric invariance	3788.28	553	< 0.001	0.986	0.071	0.060	0.988	0.001	0.001	0.001	0.001
	Scalar invariance	4463.95	626	< 0.001	0.985	0.073	0.059	0.986	0.001	0.002	0.001	0.002

df: degrees of freedom; TLI: Tucker Lewis index; RMSEA: root-mean-square error of approximation; SRMR: standardised root mean square residual; CFI: comparative fit index; Δ: change.

### Linear mixed models (Aim 3)

We identified the best model through a model selection procedure (performance score: 84.80%; see Table B of the Supplementary Materials for comparisons among models). It included the effects of gender, geographic area, emotion type, and all the interactions. In the Supplementary Materials, we reported in Table C the post-hoc tests and the descriptive statistics about the three-way interaction in Table D.

The LMM revealed a significant effect of gender, *F*(1, 2288) = 70.73, *p* < 0.001: Females (*M* = 2.79, *SD* = 1.28, 95% CI [2.76, 2.81]) had higher scores than males (*M* = 2.49, *SD* = 1.23, 95% CI [2.46, 2.52]). Also geographic area, *F*(1, 2288) = 17.75, *p* < 0.001, had a significant effect, with scores higher for European (*M* = 2.73, *SD* = 1.28, 95% CI [2.70, 2.76]) compared to non-European countries (*M* = 2.54, *SD* = 1.25, 95% CI [2.51, 2.58]). Moreover, emotion type had a significant effect, *F*(5, 11,440) = 451.41, *p* < 0.001. Post-hoc tests indicated that anger (*M* = 3.15, *SD* = 1.20, 95% CI [3.10, 3.20]) and anxiety (*M* = 3.08, *SD* = 1.17, 95% CI [3.03, 3.12]), which did not differ among them, were higher than hopelessness (*M* = 2.72, *SD* = 1.24, 95% CI [2.67, 2.77]) and hope (*M* = 2.72, *SD* = 1.14, 95% CI [2.68, 2.77])—again not differing among them. In turn, hopelessness and hope were higher than shame (*M* = 2.47, *SD* = 1.29, 95% CI [2.42, 2.52]), which was higher than pride (*M* = 1.78, *SD* = 1.07, 95% CI [1.74, 1.83]).

**Figure 2:**
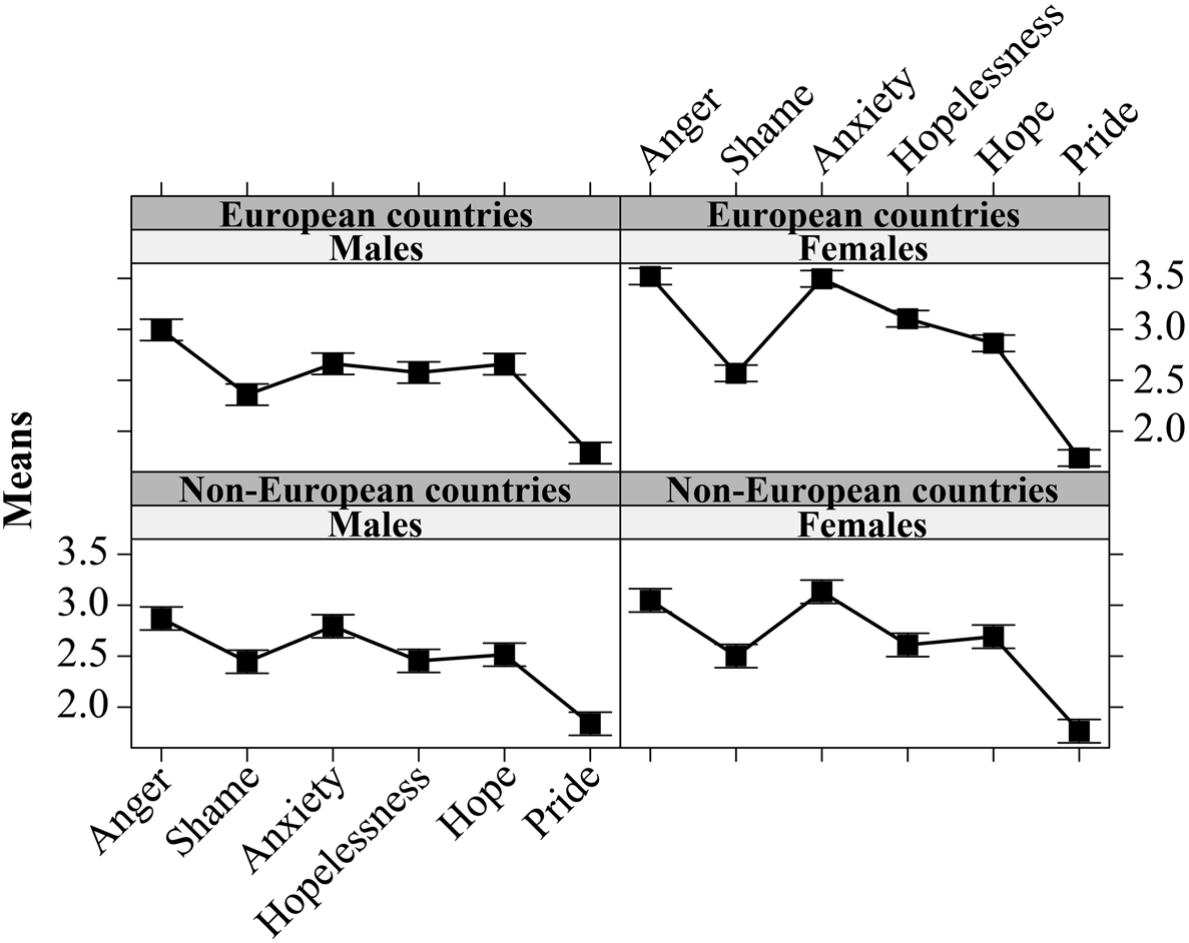
Emotion types by gender and geographic area. The bars represent the 95% confidence intervals.

These main effects were moderated by three significant two-way interactions: gender × geographic area, *F*(1, 2288) = 9.98, *p* < 0.01, gender × emotion type, *F*(5, 11,440) = 28.50, *p* < 0.001, and geographic area × emotion type, *F*(5, 11,440) = 12.37, *p* < 0.001. In turn, they were moderated by a three-way interaction, gender × geographic area × emotion type, *F*(5, 11,440) = 3.08, *p* < 0.01 (Fig. 2). Examining the post-hoc tests, we found that European females had the highest score (compared to European males, non-European males, and non-European females) for anger and hopelessness; they had higher scores than non-European females, in turn, higher than those of males, for anxiety; and they had higher scores than non-European males for hope. For shame and pride, there were no differences among the four groups.

## Discussion

As regards the relation between antecedents and coping and affective states (first aim), the SEM indicated no associations between war-related worry about education and coping. Moreover, war-related worry about macroeconomics was connected negatively with opposition and positively with support giving. War-related worry about the military domain was positively associated with both coping types. Overall, Hypotheses 1a was partially confirmed. On the one hand, it is plausible that worry about the microsystem did not provoke reactions from HE students indirectly exposed to the war simply because they perceived that their daily activities, e.g., studies, were not impacted. On the other hand, concern about military issues probably activated a coping reaction, either opposition or support. As for the impact of the macrosystem, we could argue that the higher the concern about global economic functioning, the lower the agreement with actions aimed at damaging Russia by implementing sanctions—whose effects are, however, controversial^1,5^—and the higher the propensity to help Ukrainians financially to re-establish a stable economic situation. Hypothesis 1b was partially confirmed, given that opposition was positively related to all the negative emotions (i.e., anger, shame, anxiety, and hopelessness), and support giving was positively related to hope. We also found a differential impact of the two coping types: for all the emotions except pride, there were positive links with both coping types, but they were consistently stronger for support giving compared to opposition. This delineates a general trend suggesting that HE students more inclined to help others were more emotionally engaged by aspects concerning the Ukraine war. This is coherent with previous studies indicating empathy as one of the factors associated with the tendency to provide humanitarian support, in line with the empathy-altruism hypothesis^5,34^. Moreover, the variance explained by each suggested that war-related coping markedly impacted students’ negative emotions more than positive ones. It is worth noting that the variance associated with anxiety was high. Finally, pride was characterised by a different pattern: it was higher for HE students who were more inclined to use opposition but lower for those more available to help. On the one hand, the positive relation between opposition and pride suggests the relevance of opposition strategies to reach personally valued goals, i.e., contrasting the Russian population, probably collectively identified as the invaders. On the other hand, the negative link between support-giving and pride could be explained, considering the lack of reasons for being proud when confronting the humanitarian consequences of the war.

The tested model was stable across gender, study field, and geographic area (second aim), corroborating Hypothesis 2. These data suggest that war-related worry impacts coping and emotions amongst HE students similarly across various individual and contextual characteristics, supporting the cross-cultural nature of these relations.

Finally, our findings indicated differences in the frequency HE students felt the six emotions (third aim). Anger and anxiety were the most frequent, followed by hopelessness and hope; shame and pride were the least frequent. We noted a clear pattern in emotional valence, with a greater prevalence of negative emotions over positive ones. The data revealed that, among the most frequent emotions, three out of four were prospective and future-centred, i.e., anxiety, hopelessness, and hope. Moreover, emotions were generally more frequent for females and for people living in countries closer to the war region, and these effects were further moderated by an interaction indicating that, on the whole, females from the European countries felt anger, anxiety, hopelessness, and hope more frequently. The overall prevalence of emotions for females aligns with their usual higher empathy than males^48^. Moreover, the higher emotional engagement of HE students from European countries could be explained by their shorter physical distance from the war theatre, consistent with the findings comparing mental health indicators for Ukrainian, Polish, and Taiwanese participants^13^.

## Limitations and future directions

This study had several limitations. First, we involved a convenience sample not representative of each country under study, excluding students with difficult access to the Internet or without electronic devices, and those who did not know the used languages (i.e., English or Italian). Moreover, some students completing the survey in English could have misunderstood some items, in case they were not very familiar with such language. Future research should utilise more representative samples, use preferably questionnaires available in many different languages, and implement other ways of data collection. Second, the participants were not equally distributed by gender, with a higher number of females; however, the model was strongly invariant across gender. Third, the questionnaire included self-report measures, which can be biased by social desirability; however, they constitute one of the most complete means to access mental states^49^. Future research could triangulate different measures by adding more objective indicators. Fourth, we interpreted students’ responses within the model for coping strategies; however, they could also be interpreted within other theoretical frameworks, such as those concerning intergroup attitudes^50,51^. In addition, for social support there was only one item; future research should include a larger number of items for measuring this coping strategy. Fifth, we assessed more negative than positive emotions; further studies could balance this and investigate the relationship between coping and other emotion types. Sixth, we did not explicitly investigate, for example through open questions, the antecedents of coping or emotions. Future studies could investigate the reasons underlying both constructs. Seventh, we applied a cross-sectional design, so our results are correlational. Researchers could plan longitudinal assessments to disambiguate findings concerning the reciprocal links between coping and emotions. Finally, we did not examine variations between single countries due to the reduced number of respondents for some of them. Future research could delve deeper into the effects of specific socio-cultural contexts on war-related coping and emotions.

## Applied implications

Knowledge about the relationship between war-related coping and emotions is the basis for developing preparedness and support measures to counteract the traumatic impact of wars, particularly for HE students who were indirect victims. Our findings call for evidence-based policy recommendations implemented by local, national, and international institutions to combat the negative short and long-term psychological sequelae of being witnesses of war. Understanding that war-related emotions can relate to specific response strategies, in turn, associated with worries concerning specific domains, gives an opportunity to increase awareness of the mechanisms underlying people’s responses to war. For example, helping people to rely on positive psychology is one of the ways that can contribute to transforming hopelessness into hope and diminish other war-related negative emotions, together with fostering adaptive coping responses^10^. Moreover, public campaigns can be conducted at global levels to foster awareness about the psychological processes related to wars and to disseminate first-aid self-care resources widely^10,52^. In parallel with this, acknowledging that HE students are easy targets of online misinformation and disinformation, including fake news, bots, and trolls^27,53^, educational institutions can implement actions to help them develop resources to protect themselves when retrieving information from social media.

## Method

### Participants

We used a convenience sample of 2314 HE students (*M*_*age*_ = 23.30, *SD* = 5.74; range: 18–65 years; 56% females) from 16 countries: Croatia, Ecuador, Gambia, Greece, India, Indonesia, Italy, Japan, Mexico, Pakistan, Poland, Portugal, Romania, Serbia, Slovenia, and Spain. Overall, nine countries were included within the geographical area of Europe (Croatia, Greece, Italy, Poland, Portugal, Romania, Serbia, Slovenia, and Spain), while seven countries were from the other parts of the World (Ecuador, Gambia, India, Indonesia, Japan, Mexico, and Pakistan). We excluded 381 of the initial participants because the number of respondents per country was lower than 50 and the other 244 participants because they were from Ukraine (*n* = 228) and Russia (*n* = 16).

Seventy-six percent of the students of the final sample were attending bachelor’s courses, 20% master’s courses, and 4% doctoral courses. The most frequent field of study was social sciences (51%), followed by applied sciences (27%), natural sciences (12%), and arts and humanities (10%). Most of the students were studying in their own country (96%), while a small proportion was studying abroad (3%) and on exchange (1%). The economic status was above average for 12% of them, on average for 72%, and below average for 16%. Sixty-six percent lived in urban areas, 20% in suburban areas, and 14% in rural areas. Sixteen percent had a full-time job, 35% a part-time job, and 49% did not work. See Table E of the Supplementary Materials for the descriptive statistics by each country.

As regards participants’ experience with conflicts, 5% reported being personally affected by wars, and 15% and 11% had, respectively, parents or friends affected by such situations. In addition, some students had relatives and/or friends who lived in Ukraine (10%) or Russia (12%).

### Procedure

Data collection took place through an online survey between 22 March and 22 May 2022 (the second and third month of the war), a period characterised by war-related socio-economic uncertainties for many countries. The questionnaire was initially developed in English, then translated into Italian, and released in both languages through the open-source web application 1KA (One Click Survey; http://www.1ka.si). HE students were invited to complete the survey through advertisements sent through various communication systems worldwide (e.g., email invitations and social media posts). They gave their informed consent before participating.

The study was approved by the Ethical Committee of the Department of Human Sciences of the University of Verona, Italy (protocol number 159372), and it complies with the Declaration of Helsinki regarding research on human participants.

### Measures

The questionnaire included 36 questions. Our analyses focused on six of them (comprising 26 items), measuring worry due to different war-related domains (i.e., education domain, macroeconomics domain, and military domain), war-related coping strategies (i.e., opposition and support giving), and emotions (see Table F of the Supplementary Materials for the list of the items). We also included socio-demographic information (described in the Participants section) in our analyses.

#### Worry due to war-related domains

The students were asked how worried they were about three domains: (a) The *education domain* asked about personal education (i.e., *To what extent do you worry about the personal circumstances of the Russia-Ukraine war 2022?*), using five items (e.g., *Future education*; *Student scholarships*); (b) the *macroeconomics domain* asked about the economic environment (i.e., *To what extent do you worry about the economic circumstances during the Russia-Ukraine war 2022?*), using five items (e.g., *Economic crisis*; *Increasing poverty*); and (c) the *military domain* asked about expectations about future conflicts (i.e., *To what extent do you worry about the military circumstances of the Russia-Ukraine war 2022?*), using five items (e.g., *Extending the war to my country*; *Similar military actions in the future*). The items were developed ad hoc and had to be rated on a 5-point scale (1 = *not at all* and 5 = *extremely*).

#### Coping strategies

We asked to evaluate (i.e., *Please rate your agreement with the following statements related to the Russia-Ukraine war 2022*) coping strategies using five items developed ad hoc, to be rated on a 5-point scale (1 = *strongly disagree* and 5 = *strongly agree*). Following Zimmer-Gembeck and Skinner’s^25^ taxonomy, we distinguished two dimensions concerning *opposition* (four items, e.g., *Russian tourists are not welcome in my country*; *I avoid buying Russian products*) and *support giving* (one item, i.e., *I am willing to help Ukraine with donations*).

#### Emotions

We measured the frequency (i.e., *How often do you feel the following emotions during the Russia-Ukraine war 2022?*) of four negative emotions (i.e., anger, shame, anxiety, and hopelessness) and two positive emotions (i.e., hope and pride) using a six-item adaptation of the *Achievement Emotions Adjective List* (AEAL^54^). Considering that the war began on 24 February 2022 and that the survey administration took place between 22 March and 22 May 2022, the assessment referred to the very first phases of the conflict. Items had to be rated on a 5-point scale (1 = *never* and 5 = *always*).

### Data analysis

We used the R software and its packages (Version 4.2.3^55^; see Paragraph A of the Supplementary Materials for the list of packages). First, we conducted a SEM testing both the measurement model (i.e., concerning the rules of correspondence between observed and latent variables, e.g., between the five items about education domain and the latent variable called Education domain) and the structural model (i.e., about the relations linking the hypothesised model constructs, e.g., between the latent variable Opposition and each of the six emotions). In particular, for the latter we considered the relations between war-related worry (i.e., pertaining to education, macroeconomics, and military domains) and coping (i.e., opposition and support giving), and in turn between coping and the six emotions (i.e., anger, shame, anxiety, hopelessness, hope, and pride). We used the diagonally weighted least squares estimator (DWLS, lavaan package), examining the measurement and structural models. There were no missing data. The indexes for model fit were TLI, GFI, RMSEA, SRMR, CFI, and AGFI, with TLI and GFI > 0.95, RMSEA and SRMR < 0.08, and CFI and AGFI > 0.90, as thresholds^56–59^. We also calculated McDonald’s^60^ omega (ω; see Table 1) concerning reliability of the measures (cut-off for good reliability: ω > 0.70; semTools package). In Table A of the Supplementary Materials we reported both the standardised factor loadings and the paths between the latent variables.

Second, we conducted the measurement invariance analysis^47,61,62^ by comparing the difference in the fit of a series of sequentially constrained models concerning configural (with the same latent constructs studied without imposing equality constraints across groups), metric (imposing the equality of factorial weights restriction across groups), and scalar invariance (imposing the equality of factorial weights and intercepts across groups). We examined invariance across gender (male, female), study field (social sciences, applied sciences, natural and life sciences, arts and humanities), and geographic area (European countries, other countries). Since χ^2^ is likely to be overly sensitive for large sample sizes^63^, we examined the differences between CFI values (ΔCFI) for two sequentially constrained models (cut-off values: ΔRMSEA < 0.015, ΔSRMR < 0.030 in the configural vs. metric comparison and < 0.010 in the metric vs. scalar comparison, and ΔCFI < 0.010)^64,65^.

Third, we examined the differences in emotions through a LMM (lme4, emmeans, effects, and car R packages). We performed a Type III analysis of variance table with Satterthwaite’s method^66^ and we used the Bonferroni correction for post-hoc tests (level of significance: *p* < 0.010). Initially, we used a model selection procedure to check whether the most complex models were significantly better at accounting for the observed data than the simpler models (performance package). In Table B of the Supplementary Materials, we reported the description and the indexes of the 10 models we tested. The full model comprised participants as a random effect; gender, study field, and geographic area as between-subject fixed effects; emotion type as within-subject fixed effect; all the two-way, three-way, and four-way interactions between the fixed effects; and the score of the six emotions as the dependent variable. After calculating eight fit indexes, we examined the composite performance score obtained by normalising the eight indexes and inserting the mean value for each model. The score can vary between 0 and 100%, with higher values associated with a better model performance^67,68^.

### Supplementary Information

Supplementary Information.

## Data Availability

The datasets used and/or analysed during the current study available from the corresponding author on reasonable request.
