# Supplementary material for: Coping and emotions of global higher education students to the Ukraine war worldwide
Source: Sci Rep. 2024 Apr 12;14:8561. doi: 10.1038/s41598-024-59009-3 (PMC11014932; doi:10.1038/s41598-024-59009-3)
Supplement: Supplementary file 1 — Supplementary Information. [file 41598_2024_59009_MOESM1_ESM.docx]

**Supplementary Materials**

**Paragraph A**

For running the analyses, we used the following R packages: car^1^, effects^2^, emmeans^3^, lavaan^4^, lme4^5^, performance^6^, and semTools^7^.

**References**

1. Fox, J., & Weisberg, S. (2019). *An R companion to applied regression*. Sage.
2. Fox, J., & Weisberg, S. (2018). Visualizing fit and lack of fit in complex regression models with predictor effect plots and partial residuals. *Journal of Statistical Software, 87*(9), 1–27. <https://www.jstatsoft.org/article/view/v087i09>
3. Lenth, R. V. (2021). *emmeans: Estimated marginal means, aka Least-Squares Means*. <https://CRAN.R-project.org/package=emmeans>
4. Rosseel, T. (2012). lavaan: An R package for structural equation modeling. *Journal of Statistical Software, 48*(2), 1–36. <http://www.jstatsoft.org/v48/i02/>
5. Bates, D., Mächler, M., Bolker, B., & Walker, S. (2015). Fitting Linear Mixed-Effects Models using lme4. *Journal of Statistical Software, 67*(1), 1–48. <https://doi.org/10.18637/jss.v067.i01>
6. Lüdecke, D., Ben-Shachar, M. S., Patil, I., Waggoner, P., & Makowski, D. (2021). performance: An R package for assessment, comparison and testing of statistical models. *Journal of Open Source Software, 6*(60), 3139. <https://doi.org/10.21105/joss.03139>
7. Jorgensen, T. D., Pornprasertmanit, S., Schoemann, A. M., & Rosseel, Y. (2020). *semTools: Useful tools for structural equation modeling*. <https://CRAN.R-project.org/package=semTools>

**Table A**

*Standardised Factor Loadings* *(λ), Paths Between Latent Variables (β), and Standard Errors (SE) for the Constructs Used in the SEM*

| Factor | Observed variable/Latent variable | λ/β | *SE* |
| --- | --- | --- | --- |
| Education domain | Q15b | .815*** | .006 |
|  | Q15e | .904*** | .005 |
|  | Q15f | .915*** | .005 |
|  | Q15g | .908*** | .005 |
|  | Q15h | .894*** | .005 |
| Macroeconomics domain | Q12c | .827*** | .006 |
|  | Q12d | .737*** | .007 |
|  | Q12g | .852*** | .006 |
|  | Q12h | .902*** | .006 |
|  | Q12i | .795*** | .006 |
| Military domain | Q16a | .803*** | .006 |
|  | Q16b | .813*** | .006 |
|  | Q16c | .895*** | .005 |
|  | Q16d | .878*** | .006 |
|  | Q16f | .818*** | .006 |
| Opposition | Q10a | .817*** | .011 |
|  | Q10b | .861*** | .011 |
|  | Q10c | .832*** | .010 |
|  | Q10d | .770*** | .012 |
| Support giving | Q10g | .518*** | .013 |
| Education domain | Opposition | .010 | .015 |
|  | Support giving | -.004 | .023 |
| Macroeconomics domain | Opposition | -.191*** | .019 |
|  | Support giving | .145*** | .029 |
| Military domain | Opposition | .393*** | .016 |
|  | Support giving | .575*** | .039 |
| Opposition | Anger | .170*** | .014 |
|  | Shame | .099*** | .014 |
|  | Anxiety | .175*** | .014 |
|  | Hopelessness | .128*** | .014 |
|  | Hope | .086*** | .014 |
|  | Pride | .262*** | .015 |
| Support giving | Anger | .542*** | .017 |
|  | Shame | .410*** | .015 |
|  | Anxiety | .717*** | .021 |
|  | Hopelessness | .494*** | .016 |
|  | Hope | .188*** | .011 |
|  | Pride | -.128*** | .012 |

*Note.* ****p* < .001.

**Table B**

*Indices of Model Performance for the Tested Models*

| Models | AIC weights | AICc weights | BIC weights | Conditional *R^2^* | Marginal *R^2^* | ICC | RMSE | Sigma | Performance score |
| --- | --- | --- | --- | --- | --- | --- | --- | --- | --- |
| Model 3. gender × geographic area × emotion type | 1.00 | 1.00 | .034 | .363 | .157 | .244 | 0.955 | 1.012 | 84.80% |
| Model 7. gender × emotion type | < .001 | < .001 | .966 | .359 | .150 | .246 | 0.958 | 1.015 | 70.57% |
| Model 1. gender × study field × geographic area × emotion type | < .001 | < .001 | < .001 | .368 | .166 | .242 | 0.951 | 1.008 | 60.87% |
| Model 4. study field × geographic area × emotion type | < .001 | < .001 | < .001 | .355 | .142 | .248 | 0.960 | 1.018 | 57.29% |
| Model 10. geographic area × emotion type | < .001 | < .001 | < .001 | .353 | .137 | .251 | 0.961 | 1.020 | 56.76% |
| Model 9. study field × emotion type | < .001 | < .001 | < .001 | .349 | .129 | .252 | 0.964 | 1.023 | 55.45% |
| Model 8. study field × geographic area | < .001 | < .001 | < .001 | .195 | .009 | .188 | 1.081 | 1.137 | 1.95% |
| Model 5. gender × study field | < .001 | < .001 | < .001 | .195 | .015 | .183 | 1.082 | 1.137 | 1.51% |
| Model 6. gender × geographic area | < .001 | < .001 | < .001 | .195 | .018 | .181 | 1.082 | 1.137 | 1.34% |
| Model 2. gender × study field × geographic area | < .001 | < .001 | < .001 | .195 | .022 | .177 | 1.082 | 1.137 | 1.03% |

*Note.* The symbol × indicates the interaction between parameters. Gender (male, female); geographic area (European countries, non-European countries); emotion type (anger, shame, anxiety, hopelessness, hope, pride); study field (social sciences, applied sciences, natural and life sciences, arts and humanities). AIC = Akaike Information Criterion; AICc = sample-size adjusted AIC; BIC = Bayesian Information Criterion; *R^2^* = coefficient of determination; ICC = Intra-class Correlation Coefficient; RMSE = Root Mean Squared Error. For each model, the performance score is a composite score obtained by normalising the previous eight indices and calculating their mean value. Model 1 was the full model testing the effects of the four factors, the six 2-way interactions, the three 3-way interactions, and the 4-way interaction. Models 2, 3 and 4 included the effects of the three factors, the three 2-way interactions, and the 3-way interaction. Models 5, 6, 7, 8, 9, and 10 included the effects of the two factors and the 2-way interaction. The ten models are ordered according to their performance score.

**Table C**

*F-Value (With Degrees of Freedom) and Level of Significance for Main Effects and Three-Way Interaction, and Key Bonferroni Tests and Effect Sizes (Cohen’s d). The Results Correspond to Model 3 of Table B*

| Significant effects or interactions | *F* (df) | *p* | Bonferroni post-hoc comparisons | *t* | *p* | *d* |
| --- | --- | --- | --- | --- | --- | --- |
| Gender | 70.73 (1, 2288) | < .001 | Male vs. Female | 8.41 | < .001 | 0.256 |
| Geographic area | 17.75 (1, 2288) | < .001 | European vs. Non-European countries | 4.21 | < .001 | 0.128 |
| Emotion type | 451.41 (5, 11440) | < .001 | Anger vs. Shame  Anger vs. Anxiety  Anger vs. Hopelessness  Anger vs. Hope  Anger vs. Pride  Shame vs. Anxiety  Shame vs. Hopelessness  Shame vs. Hope  Shame vs. Pride  Anxiety vs. Hopelessness  Anxiety vs. Hope  Anxiety vs. Pride  Hopelessness vs. Hope  Hopelessness vs. Pride  Hope vs. Pride | 20.16  2.43  13.98  12.42  41.67  -17.73  -6.18  -7.75  21.51  11.55  9.99  39.24  -1.56  27.69  29.25 | < .001  n.s.  < .001  < .001  < .001  < .001  < .001  < .001  < .001  < .001  < .001  < .001  n.s.  < .001  < .001 | 0.619  0.075  0.429  0.381  1.280  0.545  0.190  0.238  0.660  0.355  0.307  1.205  0.048  0.850  0.898 |
| Gender × Geographic area × Emotion type | 3.08 (5, 11440) | < .01 | Anger: Male, European vs. Male, non-European countries  Anger: Female, European vs. Female, non-European countries  Anger: Male, European vs. Female, European countries  Anger: Male, non-European vs. Female, non-European countries  Anger: Male, European vs. Female, non-European countries  Anger: Male, non-European vs. Female, European countries  Shame: Male, European vs. Male, non-European countries  Shame: Female, European vs. Female, non-European countries  Shame: Male, European vs. Female, European countries  Shame: Male, non-European vs. Female, non-European countries  Shame: Male, European vs. Female, non-European countries  Shame: Male, non-European vs. Female, European countries  Anxiety: Male, European vs. Male, non-European countries  Anxiety: Female, European vs. Female, non-European countries  Anxiety: Male, European vs. Female, European countries  Anxiety: Male, non-European vs. Female, non-European countries  Anxiety: Male, European vs. Female, non-European countries  Anxiety: Male, non-European vs. Female, European countries  Hopelessness: Male, European vs. Male, non-European countries  Hopelessness: Female, European vs. Female, non-European countries  Hopelessness: Male, European vs. Female, European countries  Hopelessness: Male, non-European vs. Female, non-European countries  Hopelessness: Male, European vs. Female, non-European countries  Hopelessness: Male, non-European vs. Female, European countries  Hope: Male, European vs. Male, non-European countries  Hope: Female, European vs. Female, non-European countries  Hope: Male, European vs. Female, European countries  Hope: Male, non-European vs. Female, non-European countries  Hope: Male, European vs. Female, non-European countries  Hope: Male, non-European vs. Female, European countries  Pride: Male, European vs. Male, non-European countries  Pride: Female, European vs. Female, non-European countries  Pride: Male, European vs. Female, European countries  Pride: Male, non-European vs. Female, non-European countries  Pride: Male, European vs. Female, non-European countries  Pride: Male, non-European vs. Female, European countries | 2.86  6.02  -7.35  -3.52  -0.06  -10.67  -0.67  0.51  -2.97  -1.43  -2.05  -2.25  -1.36  4.63  -12.33  -5.00  -6.24  -10.93  1.92  6.72  -7.81  -2.38  -0.55  -10.07  1.05  2.11  -3.10  -1.72  -0.72  -4.31  -1.14  -0.49  0.97  1.46  0.37  2.26 | n.s.  < .001  < .001  n.s.  n.s.  < .001  n.s.  n.s.  n.s.  n.s.  n.s.  n.s.  n.s.  .001  < .001  < .001  < .001  < .001  n.s.  < .001  < .001  n.s.  n.s.  < .001  n.s.  n.s.  n.s.  n.s.  n.s.  .005  n.s.  n.s.  n.s.  n.s.  n.s.  n.s. | 0.207  0.415  0.476  0.268  0.061  0.683  0.048  0.035  0.192  0.109  0.157  0.144  0.099  0.319  0.798  0.380  0.479  0.699  0.139  0.463  0.506  0.181  0.043  0.644  0.076  0.145  0.200  0.131  0.055  0.276  0.082  0.034  0.063  0.111  0.029  0.145 |

*Note.* n.s. = not significant.

**Table D**

*Means (M), Standard Deviations (SD), and 95% Confidence Intervals (CI) for Emotions, by Gender and Geographic Area*

| Variable |  | European countries |  |  | Non-European countries |  |
| --- | --- | --- | --- | --- | --- | --- |
|  | *M* | *SD* | 95% CI | *M* | *SD* | 95% CI |
| Anger |  |  |  |  |  |  |
| Male | 3.00 | 1.21 | [2.90, 3.11] | 2.79 | 1.24 | [2.69, 2.90] |
| Female | 3.48 | 1.08 | [3.41, 3.56] | 3.06 | 1.20 | [2.95, 3.18] |
| Shame |  |  |  |  |  |  |
| Male | 2.36 | 1.32 | [2.24, 2.48] | 2.41 | 1.25 | [2.30, 2.52] |
| Female | 2.55 | 1.31 | [2.47, 2.64] | 2.52 | 1.24 | [2.40, 2.64] |
| Anxiety |  |  |  |  |  |  |
| Male | 2.66 | 1.14 | [2.56, 2.76] | 2.76 | 1.16 | [2.66, 2.86] |
| Female | 3.47 | 1.05 | [3.40, 3.54] | 3.15 | 1.18 | [3.03, 3.26] |
| Hopelessness |  |  |  |  |  |  |
| Male | 2.55 | 1.28 | [2.44, 2.66] | 2.41 | 1.19 | [2.30, 2.51] |
| Female | 3.06 | 1.20 | [2.98, 3.14] | 2.59 | 1.15 | [2.48, 2.70] |
| Hope |  |  |  |  |  |  |
| Male | 2.65 | 1.07 | [2.56, 2.75] | 2.58 | 1.18 | [2.48, 2.68] |
| Female | 2.86 | 1.08 | [2.79, 2.93] | 2.71 | 1.23 | [2.59, 2.83] |
| Pride |  |  |  |  |  |  |
| Male | 1.79 | 1.03 | [1.70, 1.88] | 1.88 | 1.21 | [1.79, 1.97] |
| Female | 1.73 | 1.06 | [1.66, 1.80] | 1.76 | 1.06 | [1.66, 1.87] |

**Table E**

*Participants’ Sociodemographic Characteristics per Country*

|  | Croatia | Ecuador | Gambia | Greece | India | Indonesia | Italy | Japan | Mexico | Pakistan | Poland | Portugal | Romania | Serbia | Slovenia | Spain |
| --- | --- | --- | --- | --- | --- | --- | --- | --- | --- | --- | --- | --- | --- | --- | --- | --- |
| Number (N) | 119 | 322 | 46 | 31 | 69 | 97 | 348 | 45 | 208 | 91 | 69 | 102 | 204 | 112 | 286 | 48 |
| Age |  |  |  |  |  |  |  |  |  |  |  |  |  |  |  |  |
| *M* | 23.34 | 21.43 | 26.91 | 27.03 | 23.57 | 21.69 | 24.53 | 19.73 | 24.40 | 25.51 | 22.72 | 25.74 | 23.71 | 24.18 | 21.05 | 23.54 |
| *SD* | 4.79 | 4.22 | 6.19 | 10.32 | 3.02 | 3.51 | 6.86 | 2.73 | 6.20 | 4.16 | 3.33 | 8.09 | 6.99 | 3.55 | 3.67 | 6.54 |
| 95% CI | [22.47, 24.21] | [20.97, 21.90] | [25.08, 28.75] | [23.25, 30.82] | [22.84, 24.29] | [20.98, 22.40] | [23.80, 25.25] | [18.91, 20.56] | [23.56, 25.25] | [24.64, 26.37] | [21.92, 23.53] | [24.15, 27.32] | [22.74, 24.67] | [23.51, 24.84] | [20.62, 21.48] | [21.64, 25.44] |
| Gender |  |  |  |  |  |  |  |  |  |  |  |  |  |  |  |  |
| Male | 37.8% | 53.4% | 63.0% | 41.9% | 56.5% | 43.3% | 40.5% | 62.2% | 69.2% | 39.6% | 21.7% | 47.1% | 12.7% | 54.5% | 40.2% | 31.2% |
| Female | 62.2% | 46.6% | 37.0% | 58.1% | 43.5% | 56.7% | 59.5% | 37.8% | 30.8% | 60.4% | 78.3% | 52.9% | 87.3% | 45.5% | 59.8% | 68.8% |
| Level of study |  |  |  |  |  |  |  |  |  |  |  |  |  |  |  |  |
| Bachelor’s | 58.8% | 94.1% | 97.8% | 71.0% | 65.3% | 93.8% | 65.5% | 91.1% | 72.6% | 13.2% | 65.2% | 70.6% | 83.3% | 69.6% | 92.3% | 77.1% |
| Master’s | 41.2% | 4.3% | 2.2% | 25.8% | 27.5% | 5.2% | 27.6% | 2.2% | 18.8% | 82.4% | 34.8% | 25.5% | 14.7% | 26.8% | 7.0% | 6.2% |
| Doctorate | 0.0% | 1.6% | 0.0% | 3.2% | 7.2% | 1.0% | 6.9% | 6.7% | 8.6% | 4.4% | 0.0% | 3.9% | 2.0% | 3.6% | 0.7% | 16.7% |
| Study field |  |  |  |  |  |  |  |  |  |  |  |  |  |  |  |  |
| Social sciences | 89.1% | 27.3% | 23.9% | 9.7% | 7.2% | 74.2% | 48.3% | 84.4% | 33.7% | 30.8% | 10.1% | 38.2% | 74.0% | 36.6% | 92.0% | 70.9% |
| Applied sciences | 8.4% | 47.5% | 21.7% | 45.2% | 75.4% | 15.5% | 33.0% | 0.0% | 29.8% | 5.5% | 60.9% | 43.2% | 7.4% | 29.5% | 2.4% | 12.5% |
| Natural and life sciences | 0.8% | 17.7% | 43.5% | 41.9% | 11.6% | 4.1% | 9.8% | 2.3% | 16.3% | 19.7% | 14.5% | 10.8% | 6.9% | 21.4% | 5.3% | 8.3% |
| Arts and humanities | 1.7% | 7.5% | 10.9% | 3.2% | 5.8% | 6.2% | 8.9% | 13.3% | 20.2% | 44.0% | 14.5% | 7.8% | 11.7% | 12.5% | 0.3% | 8.3% |
| Economic status |  |  |  |  |  |  |  |  |  |  |  |  |  |  |  |  |
| Above average | 16.0% | 5.9% | 0.0% | 3.2% | 29.0% | 8.2% | 9.5% | 22.2% | 13.9% | 9.9% | 7.2% | 2.9% | 13.2% | 30.4% | 17.5% | 12.5% |
| Average | 73.1% | 69.6% | 26.1% | 74.2% | 52.2% | 85.6% | 80.5% | 66.7% | 69.7% | 63.7% | 78.3% | 74.6% | 75.5% | 60.7% | 72.7% | 77.1% |
| Below average | 10.9% | 24.5% | 73.9% | 22.6% | 18.8% | 6.2% | 10.0% | 11.1% | 16.4% | 26.4% | 14.5% | 22.5% | 11.3% | 8.9% | 9.8% | 10.4% |
| Living area |  |  |  |  |  |  |  |  |  |  |  |  |  |  |  |  |
| Urban | 64.7% | 80.3% | 47.8% | 51.6% | 88.4% | 59.8% | 70.7% | 40.0% | 73.6% | 39.6% | 68.1% | 42.2% | 78.9% | 72.3% | 44.4% | 91.7% |
| Suburban | 20.2% | 9.6% | 21.7% | 32.3% | 5.8% | 25.8% | 20.1% | 46.7% | 22.1% | 14.2% | 8.7% | 23.5% | 4.9% | 19.6% | 42.3% | 8.3% |
| Rural | 15.1% | 10.2% | 30.4% | 16.1% | 5.8% | 14.4% | 9.2% | 13.3% | 4.3% | 46.2% | 23.2% | 34.3% | 16.2% | 8.0% | 13.3% | 0.0% |
| Job |  |  |  |  |  |  |  |  |  |  |  |  |  |  |  |  |
| Full-time | 16.8% | 5.9% | 19.6% | 32.3% | 26.1% | 12.4% | 14.9% | 2.2% | 19.7% | 24.1% | 13.0% | 20.6% | 23.5% | 36.6% | 6.6% | 16.7% |
| Part-time | 42.9% | 26.4% | 21.7% | 22.6% | 29.0% | 38.1% | 31.0% | 68.9% | 41.8% | 30.8% | 26.1% | 41.2% | 26.0% | 19.6% | 53.2% | 20.8% |
| No job | 40.3% | 67.7% | 58.7% | 45.2% | 44.9% | 49.5% | 54.1% | 28.9% | 38.5% | 45.1% | 60.9% | 38.2% | 50.5% | 43.8% | 40.2% | 62.5% |

*Note.* *M* = mean; *SD* = standard deviation; CI = confidence interval.

**Table F**

*Items of the Questionnaire Examined in the Study and Response Scale*

| Factor | Item ID | Item | Response scale |
| --- | --- | --- | --- |
| Education domain |  | To what extent do you worry about the personal circumstances of the Russia-Ukraine war 2022? | 1 = *not at all*, 5 = *extremely* |
|  | Q15b | Future education |  |
|  | Q15e | Studying abroad for a longer time |  |
|  | Q15f | Student exchanges for a shorter time |  |
|  | Q15g | Student work opportunities |  |
|  | Q15h | Student scholarships |  |
| Macroeconomics domain |  | To what extent do you worry about the economic circumstances during the Russia-Ukraine war 2022? | 1 = *not at all*, 5 = *extremely* |
|  | Q12c | Higher unemployment |  |
|  | Q12d | Oversaturation of the EU labour market with cheap labour force |  |
|  | Q12g | Economic crisis |  |
|  | Q12h | Increasing poverty |  |
|  | Q12i | Increasing corruption |  |
| Military domain |  | To what extent do you worry about the military circumstances of the Russia-Ukraine war 2022? | 1 = *not at all*, 5 = *extremely* |
|  | Q16a | Further Russian military action |  |
|  | Q16b | Extending the war to my country |  |
|  | Q16c | Extending the war to other countries |  |
|  | Q16d | Similar military actions in the future |  |
|  | Q16f | The outbreak of nuclear war |  |
| Opposition |  | Please rate your agreement with the following statements related to the Russia-Ukraine war 2022. | 1 = *strongly disagree*, 5 = *strongly agree* |
|  | Q10a | I became intolerant of the Russians living in my country. |  |
|  | Q10b | Russian tourists are not welcome in my country. |  |
|  | Q10c | I avoid buying Russian products. |  |
|  | Q10d | I will avoid travelling to Russia. |  |
| Support giving |  | Please rate your agreement with the following statements related to the Russia-Ukraine war 2022. | 1 = *strongly disagree*, 5 = *strongly agree* |
|  | Q10g | I am willing to help Ukraine with donations (money, food, clothes, etc.). |  |
| Emotions |  | How often do you feel the following emotions during the Russia-Ukraine war 2022? | 1 = *never*, 5 = *always* |
| Pride | Q22a | Proud |  |
| Hope | Q22b | Hopeful |  |
| Anxiety | Q22c | Anxious |  |
| Anger | Q22d | Angry |  |
| Shame | Q22e | Ashamed |  |
| Hopelessness | Q22f | Hopeless |  |
